# Emergency laparotomy for misdiagnosed biliary cystadenoma originating from caudate lobe

**DOI:** 10.1186/1477-7819-4-76

**Published:** 2006-11-07

**Authors:** Giovanni Ramacciato, Giuseppe R Nigri, Francesco D'Angelo, Paolo Aurello, Riccardo Bellagamba, Cristina Colarossi, Emanuela Pilozzi, Massimo Del Gaudio

**Affiliations:** 1 Hepatobiliary-pancreatic Surgery, University of Rome "La Sapienza", II School of Medicine, Sant'Andrea Hospital, Rome, Italy; 2 Department of Pathology, University of Rome "La Sapienza", II School of Medicine, Sant'Andrea Hospital, Rome, Italy; 3 Department of General Surgery, Liver and Multivisceral Transplantation Unit., University of Modena and Reggio Emilia, Italy

## Abstract

**Background:**

Biliary cystadenoma is a rare benign neoplasm, which is often misdiagnosed for a hepatic abscess or a hydatid cyst that tends to recur and is at risk for progression to malignant neoplasm.

**Case presentation:**

This case describes a 30-year-old woman admitted to our institution in an emergency setting. The patient was originally misdiagnosed as affected by a hepatic hydatid cyst at another hospital, and then emergently treated at our Institution for severe abdominal pain. Histologic evaluation of the cyst showed that it was a biliary cystadenoma and, therefore, the patient underwent a hepatic resection in order to completely remove the lesion.

**Conclusion:**

Complete excision of any suspicious hepatic cystic lesion remains the best method for diagnosis and treatment of cystadenoma. Incomplete excision of most biliary cystadenoma results in a higher rate of recurrence and the risk of malignant transformation. We report this case to elucidate the clinical presentation, preoperative evaluation, and surgical treatment of these rare lesions.

## Background

Biliary cystadenomas are rare, multilocular cystic neoplasms of the liver that originate from the biliary epithelium[1]. They account for 1% of liver cystic lesions and more than 5% of symptomatic hepatic cysts[2]. They may produce massive liver enlargement, bleeding, infection, jaundice, or may obstruct the vena cava flow. Acute abdomen due to biliary cystadenoma is a rare presentation. Imaging techniques can differentiate among biliary cystadenomas and more common parasitic or simple hepatic cysts. This benign neoplasm tends to recur and they are at risk for progression to malignant neoplasm[3]. We report a case, initially misdiagnosed as hepatic hydatid cyst at another hospital, and emergently treated for severe abdominal pain. To our knowledge this is the second case in the literature originating from the caudate lobe[4]. We report the case to elucidate the clinical presentation, preoperative evaluation, and surgical treatment of this rare lesion.

## Case presentation

A 30-years-old woman was admitted to the emergency department with upper abdominal pain. The patient had been well until the day before, when she began to have pain in the epigastrium. A few hours before admission the pain, which was now localized to the upper right abdominal quadrant, became constant and severe. Anorexia and nausea developed, followed by vomiting.

The patient had a previous history of hepatic hydatid disease. Two months earlier she underwent an abdominal ultrasound for abdominal pain during admission in another hospital. Following that exam, the patient underwent magnetic resonance imaging (MRI) which was diagnostic for hepatic hydatid cyst. For this reason the patient was on mebendazole for about 2 weeks until the last hospital admission. She took an oral contraceptive for about six months in the past and was not on any other medication.

The temperature was 39°C, pulse 96 and blood pressure was 100/70. The oxygen saturation was 98 % while the patient was breathing ambient air. On physical examination the patient appeared in severe pain. Lungs and heart sounds were normal. The abdomen was distended, and bowel sounds were absent. There was tenderness in the right upper quadrant and epigastrium, with guarding and rebound tenderness. No abnormalities were found on rectal examination.

**Figure 1:**
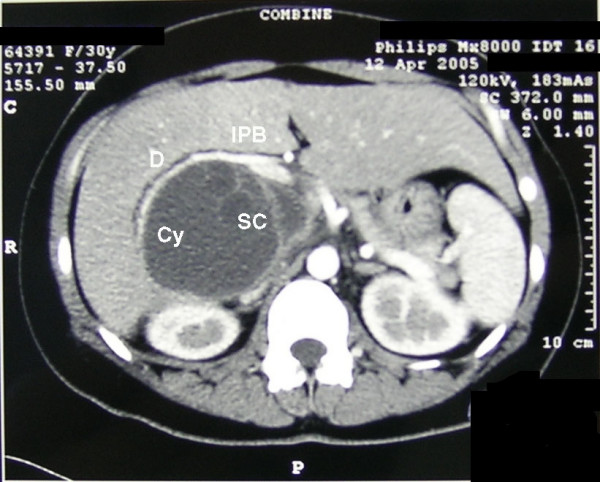
Contrast-enhanced CT scan. Cy: cyst; D: fissured cyst wall; IPB: intra-hepatic portal branch to segments VI and VII; SC: small cyst.

**Figure 2:**
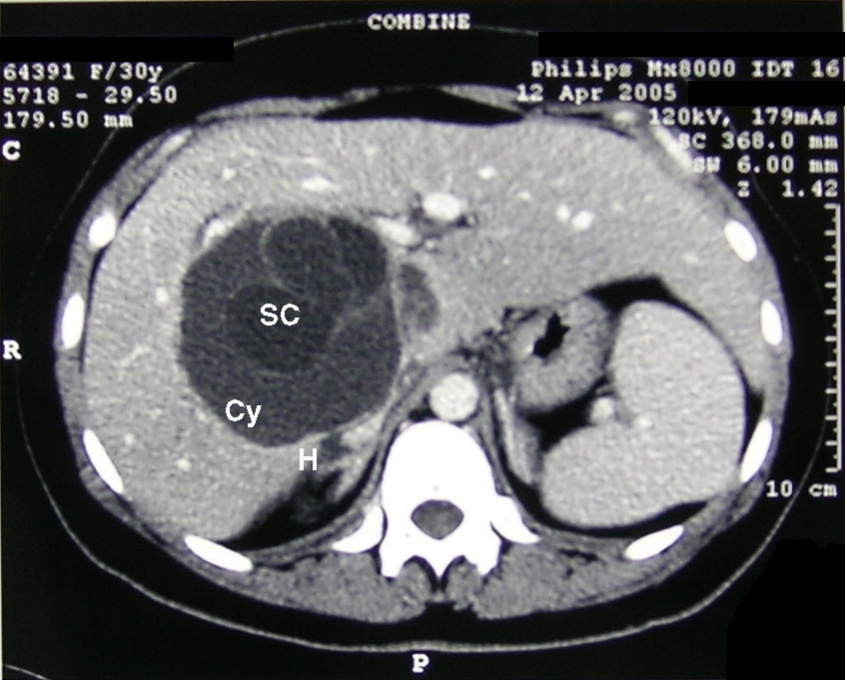
Contrast-enhanced CT scan. Cy: cyst; SC: small cysts; H: restricted hemorrhagic area adjacent to fissured cyst wall.

The hematocrit was 30%, hemoglobin 10 g/dl, red blood cells 3.3 × 10^3 ^mm^3^, white cell count 13.7 × 10^3 ^mm^3^, and neutrophils were 84.4 %. The remaining lab tests and urine were normal. Since it was an emergent setting, no further attempts were made to confirm echinococcus infestation. Abdominal ultrasound and contrast-enhanced computerized tomography (CT) scan of the abdomen showed a 9 × 11.8 × 10 cm cyst localized in the IV, VII and VIII hepatic segments, with multiple smaller cysts inside (Figure 1). The cyst was located just posterior to the portal branch for the VII and VIII segments (Figure 2). It compressed the inferior vena cava and the right and medial hepatic vein. The wall of the cyst was detached from the hepatic parenchyma as for hemorrhagic leak. Posteriorly to the hepatic peduncle, the wall of the cyst emerged at liver surface, just underneath the Glisson's capsule. Also, the adipose tissue between the liver on one side and the right adrenal gland and right kidney on the other showed signs of haemorrhage.

**Figure 3:**
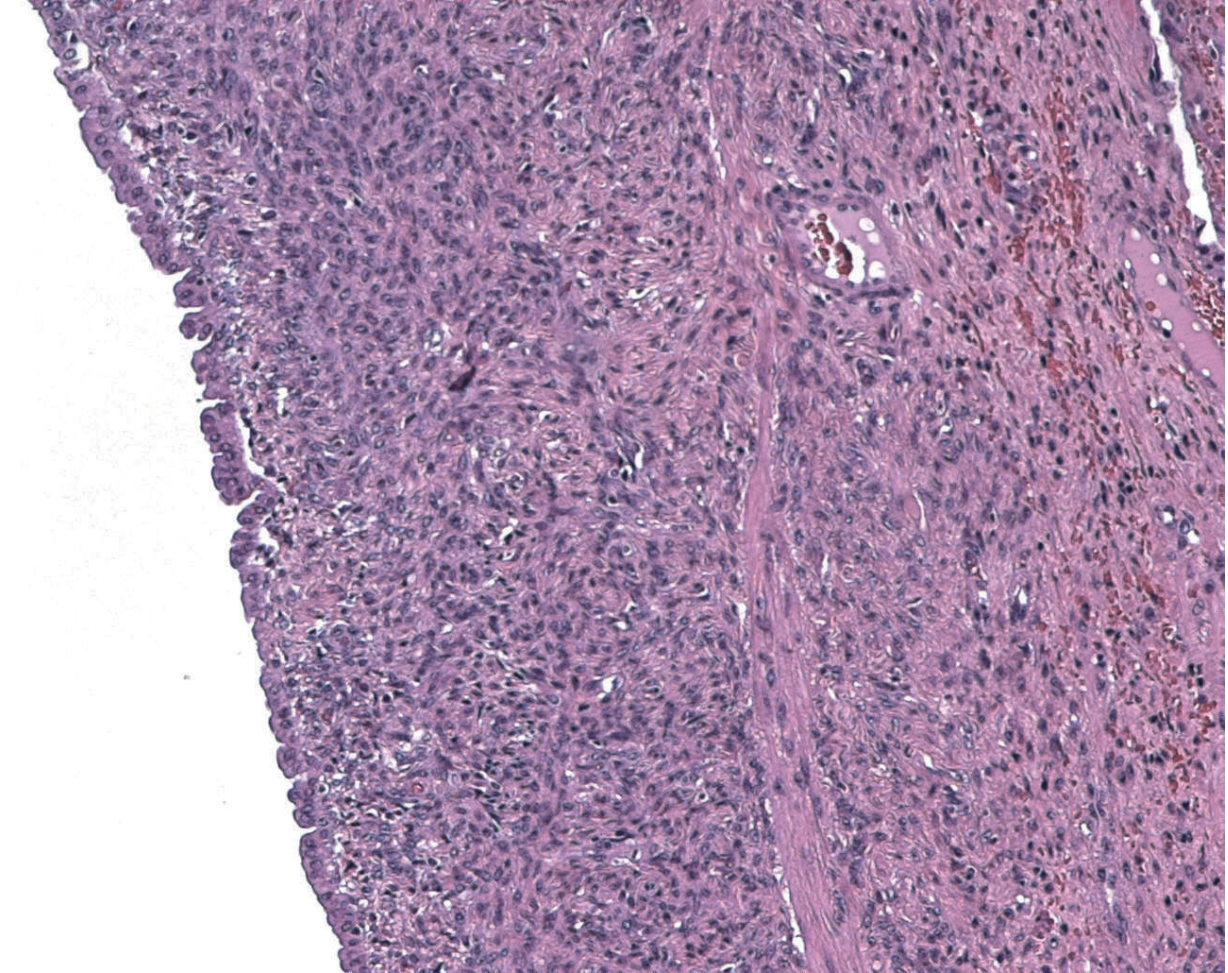
Optical microscopy (10×): the epithelium is supported by a very thick ovarian-like cellular stroma.

The patient was brought to the operating room in an emergent setting. Bilateral subcostal incision was performed. At the opening of the peritoneal cavity a small amount of clear fluid was found. Intra-operative liver ultrasound was employed. A voluminous hepatic cyst was found, which emerged to the liver surface at the level of caudate lobe and was extended up to the VIII segment. The cyst was evacuated of its content which was a brown-colored mucinous fluid. The intracystic fluid was sent for bacterial culture and evaluation for parasitic infestation. Hypertonic saline solution (33%) was injected into the cyst as a precaution in case of parasitic infestation. Partial pericystectomy was performed at the level of the caudate lobe. Ultrasound confirmed the complete evacuation of the cyst. Cholecystectomy was carried out due to the presence of biliary sludge. A biliary cystadenoma was confirmed at histologic evaluation. Sections 3 μm thick were cut and stained with hematoxylin and eosin. The sections were immunostained with primary antibodies directed against vimentin (1:100 dilution; Dako), muscle-specific actin (1:100 dilution; Dako, clone HHF35), and desmin (1:100 dilution; Dako, clone D33). Immunodetection was performed using Universal Dako Cytomation LSAB + KIT, Peroxidase. Section analysis revealed multiple communicating locules of different size. The locules were lined by columnar, cuboidal or even flattened epithelium (Figure 3). This was surrounded by a layer of highly cellular, mesenchymal tissue resembling ovarian stroma. The stromal cells were diffusely positive for vimentin and muscle specific actin, and focally positive for desmin. Cystic fluid analysis showed elevated levels of carbohydrate antigen (CA) 19.9 (126387.10 UI/ml) while serum CA 19.9, carcinoembryogenic antigen (CEA) and α-fetoprotein were within the normal range. Cultures returned negative.

Post-operative course was uneventful and the patient was discharged on post-operative day 8. After complete recovery, the patient was admitted again to our Unit for definitive treatment. Before undergoing re-operation, the patient was re-evaluated with a new abdominal CT scan which showed the partial collapse of the cyst. Patient was brought to the operating room, where she underwent caudate lobectomy and posterior segmentectomy, including segmentectomy of segments VI and VII, and atypical segmentectomy of segment IV. Intra-operative ultrasound was used to evaluate the relationship of the cyst with adjacent structures. Since a communication between the cystadenoma and the biliary tree was found, a cholangiogram followed by a single suture closure of the biliary communication was performed. Histology confirmed the previous diagnosis of biliary cystadenoma. Postoperative course was uneventful and the patient was discharged on postoperative day 8. Follow-up abdominal CT scans showed no local recurrence and the patient is doing fine 15 months after hepatic resection.

## Discussion

Biliary cystadenoma presents as an intrahepatic lesion in 90% of cases, while in the remaining cases it involves the extrahepatic biliary tree. The origin from the caudate lobe has been described only once in the literature[4]. This tumor originates from the biliary duct epithelium and more precisely from congenital aberrant bile duct. This theory is supported by the fact that this tumor has been observed as early as the first decade of life. In US the prevalence is low, with less then 200 cases reported in the literature [5-7]. The prevalence worldwide is not known. The majority of these tumors occurs in women (80–85%), and this suggested a role for hormonal influence [8-10]. The peak frequency has been reported between the fourth and the sixth decades[6,8,10]. However, in a few cases, cystadenomas have been observed in the second decade of life[11]. Macroscopically it is usually a multiloculated cyst covered by a biliary-type epithelium wall and surrounded by an ovarian-like stroma, containing smooth muscle cells[2,9]. Microscopic evaluation can easily distinguish cystadenoma from cystadenocarcinoma, based on nuclear pleomorphism and loss of nuclear stratification in the latter. A histological variant of biliary cystadenomas has been described, namely cystadenomas with mesenchymal stroma. This variant, which is more common in females, is characterized by the presence of spindle cells in the mesenchymal stroma[10]. These cells are capable of differentiating into different cell types, with a high premalignant potential. The tumor may express receptors for progesterone while histological characteristics include positivity for vimentin and cytokeratin.

Biliary cystadenomas often are diagnosed incidentally[12], during imaging studies such as ultrasound or CT scan[13]. In other cases, non specific signs and symptoms may develop, due to compression of neighboring structures, such as jaundice, signs of cholangitis, cyst infection, hemorrhage[6,8,14,15]. A rare clinical presentation, such as in this case, can be observed in an acute setting, with the characteristics of acute abdomen. In this case, the acute pain could be related to the distension on the liver capsule of the liver by the mass. The growth of cystadenoma leads to the rupture of the capsule and development of a restricted hemorrhagic area adjacent to fissured cyst wall, as shown in Fig. 2. Pain is considered the leading symptom in most series [2,14,16,17], being present in about 80% of cases[12]. Nausea, vomiting, abdominal fullness and bloating may also be present. Compression of the vena cava or the portal vein may cause lower limb edema or signs of portal hypertension such as splenomegaly. The differential diagnoses of cystic lesions of the liver include simple cysts, bilomas, hematomas, abscesses, echinococcal cysts, cystadenocarcinoma, cystic hamartomas, embryonal sarcomas, polycystic liver disease and Caroli disease. However, like in the present case, liver abscess and echinococcal cysts are the two entities most likely to be confused with biliary cystadenoma [18]. During physical examination, a palpable and tender mass in the right upper quadrant or epigastrium can be observed. Laboratory tests may show leukocytosis with a left shift in case of superinfection of the tumor. Elevation of alkaline phosphatase and bilirubin may be present. CA 19-9 may be elevated in the serum and in the cystic fluid, while CEA and α-fetoprotein levels are usually normal[6,8,19-22]. Cystic fluid analysis during laparoscopy is advocated in the surgical treatment of hepatic cysts. In fact, the presence of elevated intracystic levels of CA 19-9 can support the diagnosis of cystadenoma[1,19,20,23]. In particular, Koffron performed cystic fluid analysis in 32 patients with hepatic cysts. All patients with cystadenoma (n = 22) had elevated levels of CA 19-9, the highest found in the only patient whose cyst epithelium did not contain mesenchymal stroma. Control patients who had simple cysts (n = 8) had no significant levels of CA 19-9. Therefore, it was concluded that CA 19-9 levels can differentiate between simple hepatic cyst and cystadenoma, and that cyst fluid analysis should always be performed, allowing to spare those patients with asymptomatic simple cyst from aggressive surgical treatment.

Since biliary cystadenoma must be treated differently than most hepatic cysts, it is of paramount importance to achieve a correct diagnosis. In fact, even if the cystadenoma is a benign neoplasm, it has a high rate of recurrence and a potential for neoplastic transformation[24]. Malignant transformation rate can be as high as 30%[14]. For this reason a correct imaging study is extremely important to reduce the delay in appropriate treatment. Abdominal ultrasound and CT scan are considered the most useful radiologic studies, allowing correct diagnosis in most cases[13,25]. In particular, CT scan usually shows a multiloculated cyst, whose wall is rarely calcified. The presence of intraluminal polypoid projections originating from the wall should raise the suspicion for cystadenocarcinoma[13,26-28]. However, imaging studies are not sensitive enough to safely exclude the presence of malignant degeneration of cystadenoma[29]. Magnetic resonance imaging (MRI) can provide additional informations on the nature of the cystic fluid (i.e., serous vs. mucinous vs. hemorrhagic)[13,28,30]. Endoscopic retrograde cholangiopancreatography (ERCP), even if rarely employed, may show a cystic cavity communicating with the biliary tree[2]. Most of the authors have found fine needle aspiration (FNA) biopsy not valuable to rule out malignancy [15,18,23]. Also, if the diagnostic suspicion of hepatic cystadenoma is high, referral for surgical resection is mandatory.

The possibility for recurrence[14,17] or malignant transformation [3,31] justify an aggressive approach to cystadenoma. While aspiration, sclerosis, drainage, internal Roux-en-Y drainage, partial resection and marsupialization of cystadenoma always results in recurrence and occasional malignant degeneration, total excision of the cyst is widely supported. In the present case a two stage operation was needed, due to the acute presentation of the case and the initial misdiagnosis of the mass as an hydatid cyst. However, treatment of cystadenoma should include total excision of the tumor by standard hepatic resection [2,14,17,32-34]. It has been reported, on 15 patients, that resection of biliary cystadenoma was successfully used with rare complications and no recurrences [14]. Other authors reported no mortality or late recurrence after cystic enucleation[35]. However, since the biliary cystadenoma is often adherent to large biliary and vascular structures, enucleation must be performed with caution.

## Conclusion

Biliary cystadenoma can rarely present in acute setting. It should be expected when radiological imaging studies suggest a multilocular cystic hepatic lesion, especially in a woman. Treatment requires excision or enucleation of the cystadenoma. Hepatic resection can be necessary to achieve total excision of the cyst.

## Conflict of interest statement

The author(s) declare that they have no competing interests.

## Authors' contributions

**GR **participated in the writing process

**GN **designed the study, carried out the data and picture acquisition, drafted and revised the manuscript.

**FD, PA, RB **performed bibliographic research and participated in manuscript revision process.

**CC **and **EP **performed histologic assessment of the lesion.

**MDG **participated in the editing process.

All authors read and approved the final manuscript.
